# On the identification of hypoxic regions in subject-specific cerebral vasculature by combined CFD/MRI

**DOI:** 10.1098/rsos.220645

**Published:** 2023-01-04

**Authors:** Romana Perinajová, Pim van Ooij, Saša Kenjereš

**Affiliations:** ^1^ Department of Chemical Engineering, Delft University of Technology, Faculty of Applied Sciences, 2628 CD Delft, The Netherlands; ^2^ J.M. Burgerscentrum Research School for Fluid Mechanics, 2628 CD Delft, The Netherlands; ^3^ Department of Radiology and Nuclear Medicine, Amsterdam University Medical Centers, Location AMC, 1007 MB Amsterdam, The Netherlands

**Keywords:** CFD, MRI, oxygen transport, Alzheimer, hypoxia, brain

## Abstract

A long-time exposure to lack of oxygen (hypoxia) in some regions of the cerebrovascular system is believed to be one of the causes of cerebral neurological diseases. In the present study, we show how a combination of magnetic resonance imaging (MRI) and computational fluid dynamics (CFD) can provide a non-invasive alternative for studying blood flow and transport of oxygen within the cerebral vasculature. We perform computer simulations of oxygen mass transfer in the subject-specific geometry of the circle of Willis. The computational domain and boundary conditions are based on four-dimensional (4D)-flow MRI measurements. Two different oxygen mass transfer models are considered: passive (where oxygen is treated as a dilute chemical species in plasma) and active (where oxygen is bonded to haemoglobin) models. We show that neglecting haemoglobin transport results in a significant underestimation of the arterial wall mass transfer of oxygen. We identified the hypoxic regions along the arterial walls by introducing the critical thresholds that are obtained by comparison of the estimated range of Damköhler number (*Da* ⊂ 〈9; 57〉) with the local Sherwood number. Finally, we recommend additional validations of the combined MRI/CFD approach proposed here for larger groups of subject- or patient-specific brain vasculature systems.

## 1. Introduction

Hypoxia is a state in which an organ or tissue suffers from a lack of oxygen, which eventually leads to a cellular necrosis [1]. It is believed that this lack of oxygen can trigger mechanisms responsible for various types of pathological conditions/diseases such as dementia [2,3], atherosclerosis [4] and aneurysm generation and progression, as shown for the thoracic aortic aneurysm [5]. Circulation hypoxia is present when the oxygen is carried by red blood cells, but is not delivered to the tissue [6]. This can be caused by morphometric changes in the blood vessels, as previously demonstrated in [7,8] for stenotic arteries. Due to the presence of a stenosis, small recirculation zones are generated that are characterized by an insufficient transfer of oxygen to the arterial wall, causing a local hypoxia. Identification of these regions can provide more insights into the initial screening and potential improvements in treatment for the aforementioned pathologies. To be able to predict degree of hypoxia in blood vessels, the oxygen concentration needs to be accurately measured. Unfortunately, the present generation of measuring methods of the local oxygen concentration cannot provide the required spatial resolution or are intrusive and as such not applicable for clinical settings. Furthermore, *in vivo* tests on animal models also demonstrated a high inter-subject sensitivity, and obtained results cannot be easily generalized, as shown in [2,3]. Consequently, a new method, which will be able to provide detailed insights into oxygen mass transfer in blood vessels, needs to be introduced. Here, we propose such a method, based on a coupling between the non-invasive magnetic resonance imaging (MRI) with advanced computational fluid dynamics (CFD) including modelling of mass transfer in the cerebrovascular system. By performing such simulations, one will be able to provide a detailed mapping of the local blood flow patterns and corresponding mass transfer limitations far beyond current measurement capabilities.

In recent years, a continuous increase in computational capabilities enabled detailed numerical simulation studies of the blood flow in the complex patient-specific geometries of the cardiovascular system. To obtain subject-specific results, the four-dimensional (4D)-flow MRI measurements can be used to provide both computational domain and boundary conditions for CFD simulations [9–19]. In addition to the flow, the mass transfer needs to be modelled too. For many diseases, it is essential to understand local variations in concentration of different species, e.g. oxygen, or low-density lipoprotein (for onset of atherosclerosis) [20–22]. Few modelling approaches for oxygen mass transfer were performed either in simplified models of arteries, as in [7,8,23–30], or in the subject-specific computational geometries obtained from computerized tomography (CT) or MRI imaging techniques, as shown in [31–33]. In numerous previous computational studies of oxygen mass transfer in blood vessels, two approaches were utilized: (i) oxygen transport as a free species in plasma [7,23,26,29,30,33,34] and (ii) oxygen transport by haemoglobin [27,28,31,32,35,36]. It was suggested that the inclusion of haemoglobin-mediated oxygen transport was essential to obtain a proper representation of oxygen mass transfer in blood vessels [27].

In the present study, we propose an integral approach, which combines transport of oxygen through free diffusion in plasma with transport by haemoglobin in the subject-specific brain vascular system. The numerical simulations are based on the geometry and blood flow rates of the detailed cerebrovascular network of blood vessels obtained from 7T 4D-flow MRI scans. This enables a direct comparison between the calculated (CFD) and observed (MRI) blood flow patterns, which are important prerequisites for accurate predictions of the oxygen transport. On the basis of these simulations, we propose a method for identification of the regions within the cerebrovascular system wherein the hypoxia can take place.

## 2. Methods

### 2.1. Clinical dataset—MRI

The volunteer (male, 25 years old) underwent a 4D-flow MRI examination on a 7T MRI scanner (Achieva, Philips Healthcare, Cleveland, OH, USA) that was retrospectively gated with a peripheral pulse unit. Non-interpolated spatial resolution was 0.47 × 0.47 × 0.50 mm^3^. Echo time was 3.1 ms, repetition time was 6.8 ms and the flip angle was 20°. Velocity encoding was 150 in the *x*, *y* and *z* directions. The number of reconstructed cardiac phases was 12, resulting in a temporal resolution of 75 ms at an average heart rate of 54 beats min^−1^. The scan was accelerated with a SENSE factor of 3 in the right–left direction. Phase images were corrected for the concomitant field and eddy-current-related phase offsets. The lumen of the circle of Willis was semi-automatically segmented using commercial software (Mimics, Materialise, Leuven, Belgium). The wall shear stress (WSS) was calculated as previously described [37].

### 2.2. Computational fluid dynamics and mass transfer

#### 2.2.1. Governing equations

The conservation of mass was solved using the continuity equation under the assumption that the whole blood behaves as an incompressible fluid (*ρ* = const.),

2.1 ∇⋅u=0,

where **u** is the velocity vector, and the Navier–Stokes equations were used for solving the conservation of momentum,

2.2 $$\rho \frac{\partial u}{\partial t}+\rho (u\cdot \nabla )u=-\nabla p+\nabla \cdot (\mu \nabla u),$$

where *p* is the pressure and *μ* is the dynamic viscosity of the blood.

Oxygen transport by plasma, also known as passive oxygen transport, can be described by the dilute approximation. The convection–diffusion equation is used to describe the transport of species ‘*i*’, which can be written as follows:

2.3 $$\frac{\partial c^{\left(i\right)}}{\partial t}+\nabla \cdot (uc^{\left(i\right)})=\nabla \cdot (D\nabla c^{\left(i\right)})+R^{\left(i\right)},$$

where the last term *R*^(*i*)^ is the source/sink which is omitted for the case of passive oxygen transport. In the case of active oxygen transport (transport of oxygen bound to haemoglobin), the source/sink term describes the reaction process of binding/releasing oxygen to/from haemoglobin. There are four available locations for oxygen molecules to bind to a haemoglobin molecule, and the binding process is enhanced by already bound oxygen molecules [38]. The reactions occurring are as follows:

2.4 $$\begin{array}{rl} & \mathrm{Hb}+O_{2}\overset{K_{1}}{\to }{\mathrm{HbO}}_{2},\\ & {\mathrm{HbO}}_{2}+O_{2}\overset{K_{2}}{\to }\mathrm{Hb}{\left(O_{2}\right)}_{2},\\ & \mathrm{Hb}{\left(O_{2}\right)}_{2}+O_{2}\overset{K_{3}}{\to }\mathrm{Hb}{\left(O_{2}\right)}_{3}\\ \mathrm{and}\;\; & \mathrm{Hb}{\left(O_{2}\right)}_{3}+O_{2}\overset{K_{4}}{\to }\mathrm{Hb}{\left(O_{2}\right)}_{4}, \end{array}\}$$

with *K*_1_, *K*_2_, *K*_3_ and *K*_4_ being the reaction constants. With this approach, a total of six species need to be specified and solved in equation (2.3). This would make the calculations computationally too extensive. Instead, the oxygen–haemoglobin dissociation curve was chosen to model the process of binding. The oxygen–haemoglobin dissociation curve has a sigmoidal shape and relates the saturation of haemoglobin (*S*) to the partial pressure of oxygen (PO_2_). This can be described by Hill’s equation [39],

2.5 $$S=\frac{{\mathrm{PO}}_{2}^{n}}{{\mathrm{PO}}_{2}^{n}+P_{50}^{n}}$$

with coefficients *n* (Hill’s coefficient) and *P*_50_ (partial PO_2_ for saturation of 50%) equal to *n* = 2.7, *P*_50_ = 26.6 mmHg, respectively [8].

Several models can be used to describe the dependency between the partial PO_2_ and saturation of haemoglobin. Hill’s equation is the preferred calculation approach because of its relative simplicity. It was demonstrated that Hill’s equation predicts the actual clinical state accurately [40]. Using the oxygen–haemoglobin dissociation curve to describe active transport results in two convection–diffusion equations, which correspond to the two different species—oxygen and haemoglobin—as follows:

2.6 $$\alpha \left(\frac{\partial {\mathrm{PO}}_{2}}{\partial t}+\nabla \cdot (u{\mathrm{PO}}_{2})\right)=\alpha \nabla \cdot (D_{b}\nabla {\mathrm{PO}}_{2})+r,$$

where *α* is the solubility coefficient of oxygen, $D_{b}$ is the oxygen diffusion coefficient in blood and *r* is the rate of release of oxygen by the haem group. The transport equation for saturated haemoglobin can be written as follows:

2.7 $$[\mathrm{Hb}]\left(\frac{\partial S}{\partial t}+\nabla \cdot (uS)\right)=[\mathrm{Hb}]\nabla \cdot (D_{c}\nabla S)-r,$$

where [Hb] is the oxygen-carrying capacity of haemoglobin in blood and $D_{c}$ is the oxyhaemoglobin diffusion coefficient in blood. When combining these two equations, the reaction terms can be eliminated, and an equation for the transport of oxygen can be obtained as follows:

2.8 $$\left(1+\frac{\left[\mathrm{Hb}\right]}{\alpha }\frac{dS}{d{\mathrm{PO}}_{2}}\right)\left(\frac{\partial {\mathrm{PO}}_{2}}{\partial t}+u\cdot \nabla {\mathrm{PO}}_{2}\right)=\nabla \cdot \left[D_{b}\left(1+\frac{\left[\mathrm{Hb}\right]}{\alpha }\frac{D_{c}}{D_{b}}\frac{dS}{d{\mathrm{PO}}_{2}}\right)\nabla {\mathrm{PO}}_{2}\right],$$

where on the left-hand side of the equation coefficients in the bracket can be interpreted as a non-constant oxygen-carrying capacity, and on the right side, the coefficient can be understood as a non-constant diffusivity. The resulting equation is nonlinear, as both new coefficients are dependent on the term d*S*/dPO_2_ [8]. The computational time can be reduced further by performing the linearization of nonlinear terms in equation (2.8). After performing a first-order Taylor series expansion of equation (2.5), one can obtain

2.9 $$\frac{d\bar{S}}{d\bar{{\mathrm{PO}}_{2}}}=\frac{n\bar{S}}{\bar{{\mathrm{PO}}_{2}}}(1-\bar{S}),$$

where $\bar{{\mathrm{PO}}_{2}}$ is a reference value of the partial PO_2_ (for the highest minimization of the error, a value of 75 mmHg is used) and $\bar{S}$ is a saturation level of oxygen evaluated at $\bar{{\mathrm{PO}}_{2}}$. This equation can be used to linearize both sides of equation (2.8), yielding [8]

2.10 $$\begin{array}{rl} & \left(1+\frac{\left[\mathrm{Hb}\right]}{\alpha }\frac{n\bar{S}}{\bar{{\mathrm{PO}}_{2}}}(1-\bar{S})\right)\left(\frac{\partial {\mathrm{PO}}_{2}}{\partial t}+u\cdot \nabla {\mathrm{PO}}_{2}\right)\\ & \;=\nabla \cdot \left[D_{b}\left(1+\frac{\left[\mathrm{Hb}\right]}{\alpha }\frac{D_{c}}{D_{b}}\frac{n\bar{S}}{\bar{{\mathrm{PO}}_{2}}}(1-\bar{S})\right)\nabla {\mathrm{PO}}_{2}\right], \end{array}$$

where the coefficient on the right-hand side can be identified as the non-constant diffusivity of oxygen and the coefficient on the left-hand side as the non-constant carrying capacity of oxygen.

Finally, the non-dimensional mass transfer is evaluated through the Sherwood number, which defines the ratio between the total mass transport and purely diffusive mass transport, which is calculated as follows:

2.11 $${\mathrm{Sh}}_{w}=\frac{-d_{0}{\left(\partial c^{\left(i\right)}/\partial n\right)}_{w}}{c_{b}^{\left(i\right)}-c_{w}^{\left(i\right)}},$$

where *d*_0_ is the diameter of the artery studied, at its entry, and subscripts *b* and *w* correspond to the bulk and wall, respectively.

#### 2.2.2. Computational domain and numerical mesh

**Figure 1.**
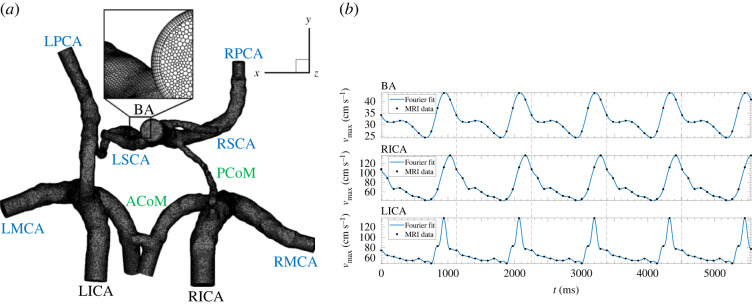
(*a*) The pre processed geometry with added outflow extensions and the polyhedral mesh created. The black labels correspond to the inlet arteries, blue to the outlet arteries and green to the connecting arteries. (*b*) The average velocity over time at the inlets. BA, basilar artery; RICA, right internal carotid artery; LICA, left internal carotid artery.

The simulations were performed on the geometry of the circle of Willis (CoW). The results of the preprocessing and meshing of the CoW geometry are shown in figure 1*a*. The arterial system studied consists of three inlets: basilar artery (BA) and left and right internal carotid artery (L(R)ACA); six outlets: left and right middle cerebral artery (L(R)MCA), left and right posterior cerebral artery (L(R)PCA) and left and right superior cerebellar artery (L(R)SCA); and two connecting arteries: ACoM and PCoM. For the geometry manipulation (which includes smoothing, opening ends and adding extensions), the open-source software, Vascular Modeling Toolkit, was used [41].

We have opted for a polyhedral mesh to perform our simulations with boundary-layer refinement. In total, 10 boundary layers were created, with the first element width of approximately 5 × 10^−3^*D*_0_ and the total width of approximately 7 × 10^−2^*D*_0_ (in BA), where *D*_0_ is the diameter of the inlet of BA. The final polyhedral grid used for the simulations is shown in figure 1*a* with the details of the boundary-layer refinement. This mesh was proven to be adequate for the simulations, as shown in the mesh-dependency analysis in the electronic supplementary material. To perform the mesh sensitivity study, a finer and a coarser mesh were created. The coarse mesh consisted of approximately 1.3 million polyhedra (initially 5 million tetrahedra), whereas the medium mesh consisted of approximately 3 million polyhedra (initially 12 million tetrahedra), and finally, the finer mesh included approximately 7.5 million polyhedral cells (initially 40 million tetrahedra). In our previous work, in which we addressed the transport of the momentum and magnetic drug carriers in the brain vascular system, a similar numerical mesh resolution proved to be sufficient to obtain grid-independent solutions [42]. The mesh sensitivity analysis of wall shear stress shows small differences between the medium and finer mesh in both the local trends and the global averages. The global averages of wall shear stress (WSS ≡ *τ*_*w*_ = −*μ*(∂*u*_‖_/∂*r*_*n*_)_wall_) were found to be 3.66 and 3.71 Pa for the medium and fine mesh, respectively. Furthermore, the grid convergence index study for a zoom-in section of the CoW, together with comparison of the locally extracted WSS profiles, is presented in the electronic supplementary material, confirming that the results obtained on the medium mesh are grid independent.

#### 2.2.3. Boundary conditions

The rigid wall assumption was made. At the wall, the no-slip condition was applied, and the concentration of oxygen was assumed to be zero. Three inlets are present in the subject-specific geometry of the circle of Willis: basilar artery (BA), left internal carotid artery (LICA) and right internal carotid artery (RICA). The velocities for each of the arteries mentioned were measured for one cycle (1034 ms) every 94 ms by the four-dimensional MRI measurement technique. An appropriate curve was subsequently fitted to the velocity data points obtained. Fourier series predicts that each curve which shows some periodicity can be decomposed into a sum of sine and cosine functions. MRI data with fitted curves are shown in figure 1*b*. The numbers obtained for harmonics used to describe the inlets of the arteries are: BA (*n* = 5), RICA (*n* = 8) and LICA (*n* = 12). The *n*-values were chosen such that the statistical term *R*^2^ (squared residuals) was equal to 1.00.

The time-dependent curves were implemented as an inlet boundary condition, and a parabolic velocity profile was constructed. The outlet mass flow rates were specified by Murray’s Law based on the conservation of mass at the branching points of arteries [43,44]. It can be written as follows:

2.12 $$\frac{q_{0}}{q_{D_{i}}}={\left(\frac{R_{0}}{R_{D_{i}}}\right)}^{1/3},$$

where *q*_0_ stands for the volumetric flow through a mother artery of radius *R*_0_, and $q_{D_{i}}$ is the volumetric flow through one of the branches with radius $R_{D_{i}}$. The resulting ratios of the total mass flow rate are as follows: LACA—12%, LMCA—25%, LPCA—9%, LSCA—5%, RACA—11%, RMCA—29%, RPCA—7% and RSCA—2%.

#### 2.2.4. Materials and physical set-up

The physical properties of the fluid (blood) are specified as follows: density of 1060 kg m^−3^, haematocrit level of 45% and non-Newtonian viscosity behaviour defined by the Carreau–Yasuda model [45],

2.13 $$\mu_{\mathrm{eff}}(\gamma )=\mu_{\infty }+(\mu_{0}-\mu_{\infty }){\left(1+{\left(\lambda \gamma \right)}^{a}\right)}^{\left(n-1\right)/a},$$

where *μ*_0_ is the viscosity at zero shear rate, *μ*_∞_ is the viscosity at infinite shear rate, *λ* is a relaxation time, and *n* and *a* are the power indices. The estimated parameters are based on fitting measured viscosity of blood for the haematocrit level of 45% [46]: *μ*_0_ = 213.1 mPa s, *μ*_∞_ = 3.14 mPa s, *λ* = 50.2 s, *n* = 0.331 and *a* = 0.8588.

For the passive transport of oxygen, a characteristic value of the Schmidt number of Sc = 2917 was used. The oxygen-carrying capacity [Hb] is specified as 0.2 ml O_2_ ml^−1^ blood, where the following properties were used: the oxygen solubility *α* = 2.5 × 10^−5^ ml O_2_ ml^−1^ plasma mmHg^−1^, the oxygen diffusion coefficient in blood $D_{b}=1.5\times 10^{-9}\;m^{2}\;s^{-1}$ and the oxyhaemoglobin diffusion coefficient in blood $D_{c}=1.5\times 10^{-11}\;m^{2}\;s^{-1}$ [8].

#### 2.2.5. Numerical set-up

In this study, the second-order upwind discretization scheme for convective terms in transport equations was applied, which ensures higher stability and accuracy of the results [47]. The solution methods used in this study for all the models mentioned here are as follows:

— solver—pressure based,
— pressure–velocity coupling—SIMPLE,
— spatial discretization,
  (i) gradient—least squares cell based,
  (ii) pressure—second order,
  (iii) momentum—second-order upwind, and
  (iv) species—second-order upwind,
— time discretization—second-order implicit, and
— residuals (continuity, velocity components, pressure and O_2_)—10^−5^.

### 2.3. Post-processing and data analysis

In total, five cycles of simulations were performed (as shown in figure 1*b*), and only the last cycle was used for the further analysis. To analyse the blood flow and oxygen mass transfer, we have calculated several additional parameters. First, the wall shear stress (*τ*_*w*_) was calculated as follows:

2.14 $$\tau_{w}=\mu \cdot \dot{\gamma_{w}},$$

where *μ* is the dynamic viscosity of the blood and $\dot{\gamma_{w}}$ is the shear rate at the wall. For comparison purposes between CFD and MRI, we have normalized *τ*_*w*_ using the respective maximal values of wall shear stress as follows:

2.15 $${\left(\tau_{w}^{i}\right)}^{*}=\frac{\left(\tau_{w}^{i}\right)}{{\left(\tau_{w}^{i}\right)}_{\max}},$$

where *i* denotes MRI or CFD. In addition, we have calculated the time-averaged quantities for *τ*_*w*_ and *Sh* as follows:

2.16 $$\mathrm{TAWSS}=\frac{1}{T}\int_{0}^{T}|\vec{\tau_{w}}|\;dt$$

and

2.17 $$\mathrm{TASh}=\frac{1}{T}\int_{0}^{T}|{\mathrm{Sh}}_{w}|\;dt,$$

where *T* is the period of a cardiac cycle, $\left|\vec{\tau_{w}}\right|$ is the absolute value of shear stress vector at the wall, and $\left|{\mathrm{Sh}}_{w}\right|$ is the Sherwood number. To identify the level of oxygen consumption in the wall, we have calculated the Damköhler number (Da), which is defined as follows:

2.18 $$\mathrm{Da}=\frac{\dot{Q}Td}{KDP_{b}},$$

where $\dot{Q}$ is oxygen consumption rate of arterial tissue, *T* is the arterial wall thickness, *d* is the mean arterial diameter, *K* is Henry’s constant, D is the oxygen diffusion coefficient and *P*_*b*_ is the bulk PO_2_ [38]. We have adopted the following values of the transport coefficients, $\dot{Q}/KD=1.96\times 10^{5}\;\mathrm{mmHg}\;{\mathrm{cm}}^{2}$ and *P*_*b*_ = 90 mmHg, in accordance with [25,48].

Finally, if any variables (*ϕ*) were compared, we have calculated the relative difference (Δ*ϕ*) as follows:

2.19 $$\Delta \phi =\frac{\phi_{\mathrm{MRI}}-\phi_{\mathrm{CFD}}}{0.5\cdot (\phi_{\mathrm{MRI}}+\phi_{\mathrm{CFD}})}\times 100\;\mathrm{in}\;(\text{\%}),$$

where *ϕ*_MRI_ and *ϕ*_CFD_ are the values of the compared variable for MRI and CFD, respectively. To be able to make more objective comparisons, the CFD results are mapped to the MRI mask by applying the following steps:

— CFD results were interpolated onto a grid with an equal cell size of 0.001 mm—to eliminate the high cell density close to the wall.
— For the voxel-to-voxel comparison, CFD mask was registered on the MRI mask.

Afterwards, the percentage difference was calculated as shown in equation 2.19.

## 3. Results

### 3.1. Blood flow validation in CoW

**Figure 2.**
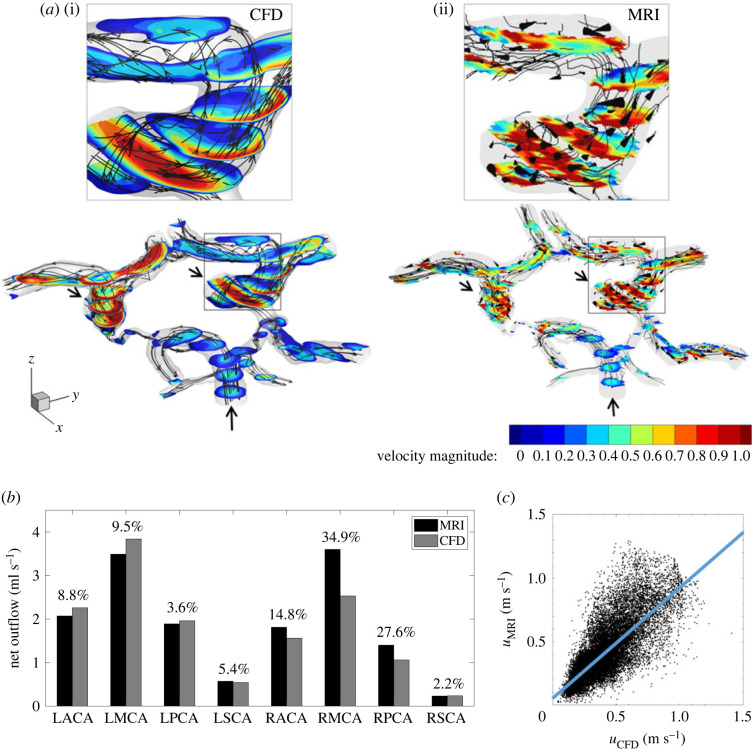
Contours of velocity magnitude at the peak systole as obtained from CFD (i) and MRI (ii) details of one of the bifurcations (*a*), comparison of flow rate in ml s^−1^ at all of the outlet faces of the circle of Willis for CFD and MRI with the estimated relative difference between the MRI and CFD of the outlet net flow rate (*b*), and the correlation plot of velocity magnitude between CFD and MRI for 10 randomly chosen *Z*-slices (out of 40 total), where the calculated *R* coefficient is equal to 0.79 (*c*).

A comparative assessment of blood flow at the systole, obtained from CFD and MRI, is shown in figure 2*a*. For both cases, we have plotted the stream traces and contours of the velocity magnitude. The details of the flow in the LICA bifurcation are shown in the highlighted regions. Figure 2*b* shows the net volumetric flow for each of the outlets as acquired by MRI (black) and calculated by CFD (grey). In addition, we have calculated the relative percentage difference in the outflows between the two methods, figure 2*b*. To statistically evaluate the agreement between the calculated and measured velocity magnitude of the blood flow in the subject-specific CoW, the voxel-to-voxel correlation of the datasets is shown in figure 2*c*. In this plot, we have selected 10 horizontal slices (along the *z*-direction) and performed extrapolation of the CFD data to the corresponding MRI mask. The Pearson product–moment correlation coefficient was calculated to be 0.79, which implies a good correlation between CFD and MRI.

**Figure 3.**
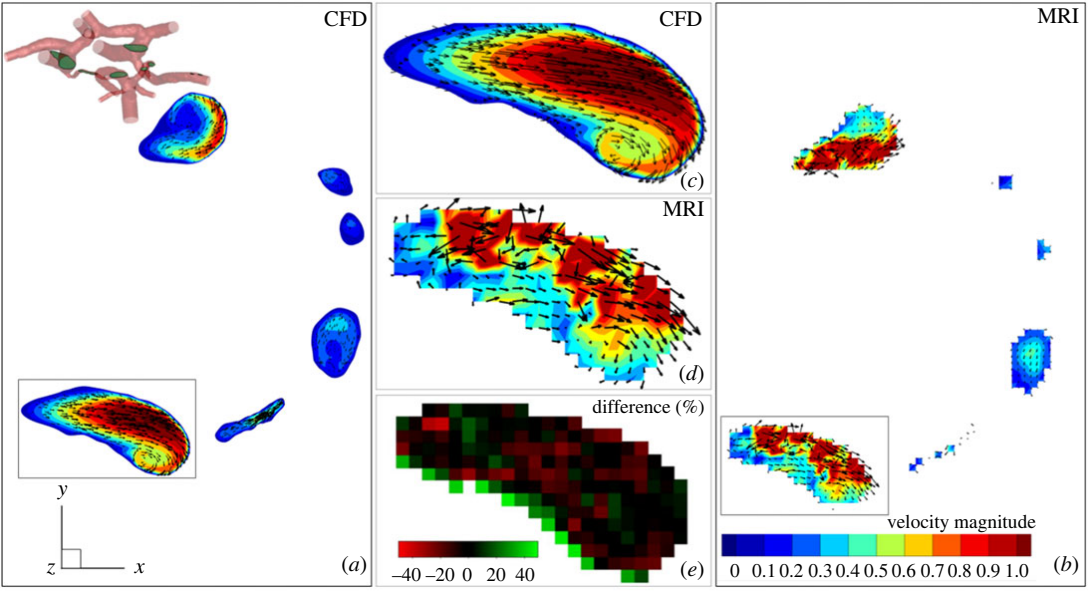
Velocity magnitude with in-plane velocity vectors for a characteristic horizontal cross-section of the circle of Willis: the results of CFD (*a*) and MRI (*b*) with the details of one of the arteries is shown for both CFD (*c*) and MRI (*d*) and the percentage difference between MRI and CFD (*e*).

We provide a more detailed comparison between the CFD and MRI results by zooming into a characteristic horizontal cross-section shown in figure 3. This cross-section shows parts of the RICA and LICA along with the small fraction of the PCoMs. The contours of the velocity magnitude are superimposed with in-plane velocity vectors. By comparing the overall agreement between the CFD (*a*) and MRI (*b*), it can be seen that the most salient flow features are captured by both methods. A zoomed-in selection (LICA) is shown in figure 3 for CFD (*c*) and for MRI (*d*) together with a voxel-to-voxel relative percentage difference between the two methods (*e*). To calculate this, we have interpolated the CFD data on the MRI grid and calculated a voxel-to-voxel difference between the two datasets, figure 2.

### 3.2. Wall shear stress validation in CoW

**Figure 4.**
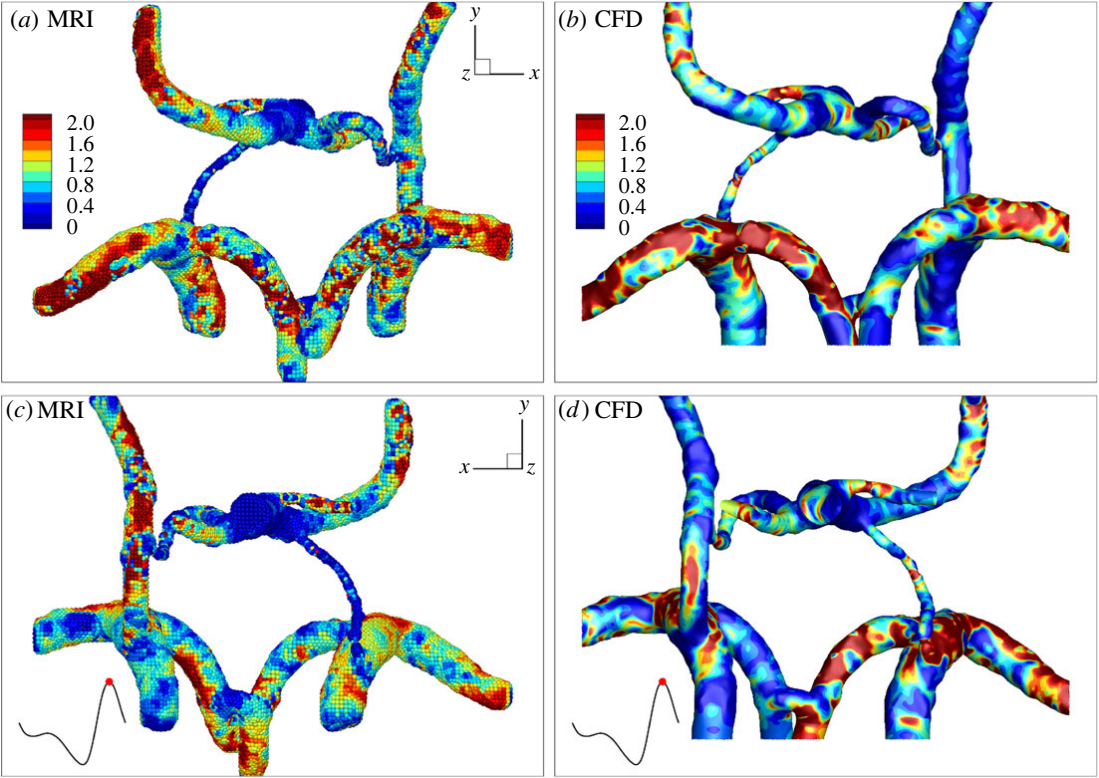
Normalized wall shear stress (WSS/WSS_mean_) at the peak systole as obtained from (*a*,*c*) MRI and (*b*,*d*) CFD from two perspectives (*a*,*b*/*c*,*d*); WSS was normalized for both cases separately by the corresponding mean value.

The non-dimensional WSS for MRI and CFD at the peak systole, for two characteristic views (the front view (*a*,*b*) and the back view (*c*,*d*)), are shown in figure 4. Note that the values were made dimensionless for each case separately with the respective mean values. To perform comparisons between MRI and CFD more quantitatively, values of the overall maximal and averaged values of WSS for three distinct time instants of the pulsating cycle were selected (*t* = *k* · *ω*, beginning of diastole; *t* = 0.6*k* · *ω,* end of diastole; 0.9*k* · *ω*, peak systole), and results are shown in table 1.

**Table 1.** Global maximal and mean values of the WSS for MRI and CFD results, for three characteristic times of a *k*th cardiac cycle (*k* · *ω*), where 0.6*k* · *ω* represents the diastole and 0.9*k* · *ω* the systole, *k* = 0, 1, 2, …

	maximal WSS			average WSS		
time (ms)	MRI	CFD	difference (%)	MRI	CFD	difference (%)
*k* · *ω*	18.66	84.63	127.74	2.89	4.61	45.87
0.6*k* · *ω*	21.72	23.24	6.76	1.81	2.04	11.95
0.9*k* · *ω*	17.82	87.62	132.40	3.97	7.42	60.58

### 3.3. Mass transport of oxygen in CoW

**Figure 5.**
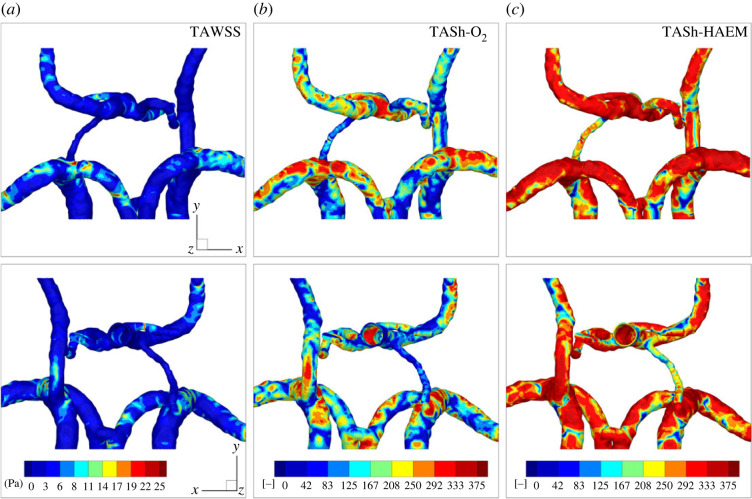
TAWSS (*a*), time-averaged Sherwood number for passive transport (TASh-O_2_) (*b*) and active transport (TASh-HAEM) (*c*).

To find a possible correlation between the momentum (flow) and mass (oxygen) transfer over the entire cardiac cycle, the time-averaged wall shear stress (TAWSS) and time-averaged Sherwood number (TASh) are calculated for both the passive and active oxygen transfer. They are visualized in figure 5, for both investigated modes of oxygen transport (passive O_2_ and active HAEM).

**Figure 6.**
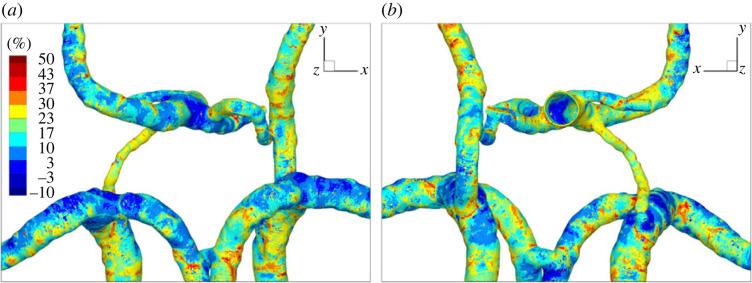
Relative percentage difference in the Sherwood number between the passive and active model from the (*a*) front and (*b*) back.

To perform a more detailed comparison between the passive and active mass transfer of oxygen, the difference between the two models is calculated for each control volume and shown in figure 6 (for two perspective views—from front (*a*) and from back (*b*)). Note that this difference was calculated similarly to equation 2.19.

### 3.4. Identification of hypoxic regions

A possible way to identify hypoxia is to compare consumption of oxygen within the wall with the mass transport from the luminal side. The consumption of oxygen within the wall can be characterized by the non-dimensional Damköhler number (Da) as defined in equation (2.18). The estimated values of Da for different regions in CoW are listed in table 2 together with the averaged diameter of the corresponding arteries (*d*_ave_) and the respective estimated wall thickness (*T*).

**Figure 7.**
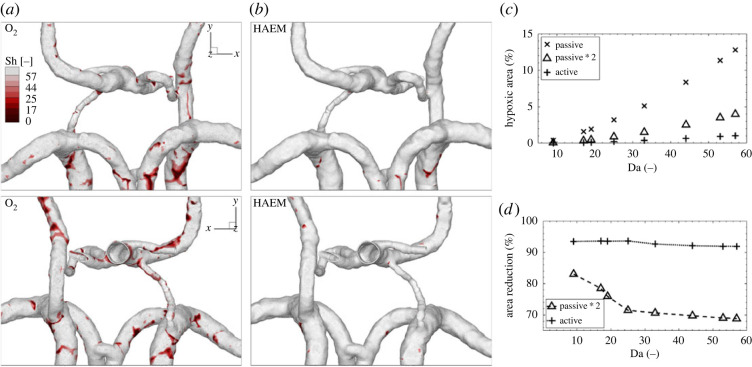
Comparison of the hypoxic areas for both oxygen mass transport models—as dissolved species (O_2_) (*a*) and as bonded to HAEM (*b*) for the boundary condition at the wall of 0 mmHg. The hypoxic regions (when Sh < Da) are shown in red, the percentage of the arterial surface area with hypoxic behaviour based on the passive, two times passive, and active oxygen transport models (*c*) and the hypoxic area reduction in comparison with the passive model for the range of the Damköhler number (*d*).

In theory, hypoxia occurs if the mass transport towards the wall (defined by Sherwood number, Sh) is lower than the consumption of oxygen in the wall (defined by Damköhler number, Da)—hence, if the condition Sh < Da applies. In figure 7, we highlight the regions of CoW, where Sh < Da. The colour range is adjusted according to the estimated values of Da (table 2). We show the hypoxic regions for the passive oxygen transport (O_2_) in (*a*) and for the active oxygen transport (HAEM) in (*b*). In addition, figure 7*c* shows the percentage of the total surface area of CoW potentially affected by hypoxia for both modes of transport (passive and active) and for passive × 2. Finally, we show the reduction (in %) of hypoxic area in respect to the passive transport for passive × 2 and active oxygen transport, figure 7*d*.

**Table 2.** Average diameter (*d*_ave_), estimated wall thickness (*T*) and calculated values of the Damköhler number (Da) for all major arteries of CoW (ICA, internal carotid artery; BA, basilar artery; ACA, anterior choroidal artery; MCA, middle cerebral artery; PCA, posterior cerebral artery; PcoM, posterior communicating artery; AcoM, anterior communicating artery; SCA, superior cerebellar artery). Note that estimates of the arterial wall thickness were based on [49].

artery	*d*_ave_ (mm)	*T* (mm)	Da (–)
ICA	4.00	0.66	57
BA	4.00	0.61	53
ACA	2.50	0.45	25
MCA	3.50	0.58	44
PCA	3.00	0.51	33
PcoM	1.00	0.39	9
AcoM	1.00	0.39	17
SCA	2.00	0.44	19

## 4. Discussion

In this work, we have investigated the blood flow and oxygen mass transfer in the circle of Willis, an integral part of the cerebrovascular system. The simulations were based on 4D-flow MRI. The measurements were used to define the computational domain and the inlet boundary conditions. In addition, we have also validated the simulated blood flow with the 4D-flow MRI measurements. For oxygen mass transfer, we have used and compared two different models of oxygen transport—passive transport (free diffusion in plasma) and active transport (combined diffusion in plasma and bound to haemoglobin). Finally, we proposed a method to identify the hypoxic regions, based on the ratio between oxygen mass transfer towards the wall and the estimated consumption by the wall.

### 4.1. Agreement in flow field

To validate the simulated results, we have compared the calculated flow field with the 4D-flow MRI measurements, figures 2 and 3. The velocity distribution within the CoW obtained from CFD is in a qualitatively reasonable agreement with MRI results. At the same time, some significant deviations in particular parts of the CoW can also be observed. The inlet region of the ICAs from CFD shows that the flow field is fully developed. However, MRI measurements do not show this behaviour. The difference between the two is a consequence of the CFD simulation having a parabolic velocity profile as the inlet condition. In reality, the velocity profile is often not fully developed, and it can change during the cardiac cycle. Consequently, the assumption of a fully developed parabolic velocity profile used at the inlet can produce differences compared with the MRI. On the other hand, the real velocity profiles based on the characteristic pulsating Womersley solution (with values of the Womersley number estimated here as $\alpha =D\sqrt{\omega /\nu }$ to be between 1 and 3 [50,51]) are similar to the parabolic solutions, and some deviations are mainly present for the ICAs, which can partially explain the differences discussed earlier between MRI and CFD in the proximity of the inlet.

To provide even more detailed comparisons between MRI and CFD, we extract a single location, as indicated in figure 3*c*–*e*. It can be seen that reasonable agreement between CFD and MRI is obtained for this particular location with a maximal absolute difference of 40%. It is noted that the maximal deviation is (as expected) obtained in the near-wall region because of the limited spatial resolution of MRI and boundary-layer resolving numerical mesh used in CFD. Additional comparison between MRI and CFD is illustrated in figure 2*b*, where the characteristic outlet flow rates (with eight outlets in total) are shown. Again, it can be seen that overall good agreement between CFD and MRI is obtained for the majority of outlets, with the exception of the RMCA and RPCA where CFD calculation predicts 35% and 28% lower flow rates, respectively.

### 4.2. Agreement in wall shear stress

To properly capture WSS, a very high resolution close to the aortic wall is necessary. It has been shown that MRI is not able to capture the velocity gradient properly, and the absolute values of WSS are often underestimated by MRI [52,53]. This can be observed also in our data. For peak systole (*t* = 0.9 *k* · *ω*), the difference between maximal WSS of MRI and CFD is very large. In contrast, at the diastole (*t* = 0.6*k* · *ω*), agreement is much better with less than 10% difference. These trends are similar also for the averaged WSS, although with significantly smaller deviations. The most significant deviation is again observed at the peak systole with a difference of 60%. Both maximal and averaged values of WSS obtained from MRI show consistently lower values compared with CFD.

While the absolute values of WSS are generally underestimated by MRI, the local distributions of high/low WSS can still be properly estimated by this technique [19]. To be able to compare the WSS distribution between CFD and MRI, we have normalized both of the datasets by their respective average values. This allows for a qualitative comparison between the two methods [19]. As shown in figure 4, the local WSS distribution in the different arteries of CoW is similar for MRI and CFD. However, the agreement between measured (from MRI) and numerically calculated (from CFD) WSS is significantly less satisfactory in comparison with the velocity magnitude. Generally, the decreased agreement is expected, since estimation of the WSS requires very accurate calculation of the velocity gradients in the proximity of the arterial wall. Here, we postulate that one of the primary reasons for this difference is due to the different spatial resolutions of MRI and CFD. In contrast to a uniform spatial resolution of MRI, the CFD spatial resolution is significantly refined in the proximity of the arterial wall to properly resolve the hydrodynamic (momentum) boundary layers, which will lead to more accurate calculations of the velocity gradients and, consequently, to more accurate WSS.

### 4.3. On modelling of the oxygen mass transfer

It can be seen that at many locations with the high values of TAWSS, the TASh distributions also reach their local maximum. The contour plots of the TASh number for passive (O_2_) and active (HAEM) oxygen transport exhibit qualitatively similar behaviour, with the latter showing at first sight significantly higher values in almost the entire CoW, i.e. the consequence of an increase of the effective diffusivity of the oxygen for the active model.

The difference in Sh between passive and active oxygen mass transfer indicates the significance of active oxygen mass transfer, i.e. the importance of the haemoglobin coupling in oxygen mass transport modelling. It can be seen that this difference shows a strong local dependency, with values between +50% and −10%, and with the highest difference in regions characterized by a low Sherwood number. On the basis of this distribution, we can also conclude that there is no simple pre-factor scaling of the passive oxygen mass transfer, such as would be able to match the active mass transfer of oxygen locally.

### 4.4. Identification of hypoxia

The low values of Sherwood numbers are associated with regions of a low wall mass transfer of oxygen, which potentially can lead to hypoxia. To identify hypoxic regions at the arterial wall, we will use the criterion that the local rate of oxygen consumption is higher than the local Sherwood number [32,54]. The non-dimensional rate of oxygen consumption can be expressed as Damköhler number (Da), which is defined in equation (2.18).

In the present study, the calculated averaged mean diameters of the major arteries and their estimated wall thickness are given in table 2. Note that the estimate of the arterial wall thickness was based on the study of Harteveld *et al.* [49]. It can be seen that the potential hypoxic regions are significantly reduced in the case of active oxygen transport. The comparison between the percentage of the arterial wall surface affected by hypoxia for passive and active mass transfer of oxygen for different values of Da is shown in figure 7*c*. Some studies in the literature suggest that the simplified calculation of the oxygen mass transfer based on the passive model can be sufficient when the final results of the non-dimensional wall mass transfer were multiplied by a factor of two, which provided a good agreement with the results of the active model [25]. This is the reason why we also included results of the passive model multiplied by a factor two in figure 7*c*. It can be seen that the percentage of the hypoxic area increases with Da, and that maximum values of 13%, 4% and 1% are obtained for the passive, passive scaled twice and active oxygen models, respectively. In addition, we calculated the hypoxic area reduction as predicted by the active and twice passive models compared with the passive oxygen transfer model, and summarized results are given in figure 7*d*. Values of around 70% were obtained for the majority of big arteries for the twice passive oxygen transfer model, whereas values of approximately 90% were obtained for the active oxygen transfer model. It can be concluded that a simple pre-scaling by a factor of two cannot provide a close agreement with the active oxygen transport model. This also additionally confirms the need to apply a more advanced active model of oxygen transport to identify possible hypoxic regions within the subject- or patient-specific cerebral vascular system.

Finally, we need to comment on the validity of the identified hypoxic regions. While we were able to identify a considerable percentage of the total surface area to be hypoxic, the data might have been affected by the uncertainty brought to the simulations due to the relatively low resolution of 4D-flow MRI. Similarly as shown for WSS variability due to segmentation in the aorta [53], variability in Sh number could be expected as well and should be evaluated on a larger cohort including intra- and inter-observer analysis. However, the proposed method for identification of hypoxia in the cerebrovascular system shows a potential to be used in further studies.

### 4.5. Limitations

Several assumptions were made in this study, and therefore, the pertinent limitations of the models have to be mentioned. Regarding blood flow simulations, an initial attempt was made to match the CFD outflows at the outlet faces so as to be as close as possible to the measured MRI values. Unfortunately, due to limited MRI resolution, the measured velocity field does not comply with the overall divergence-free condition in CFD (i.e. conservation of mass was not satisfied), which resulted in a compromising approach where Murray’s Law was applied to calculate the flow rates at the outlets. In addition, we have made an assumption of rigid wall. In reality, compliance of arteries allows the wall to move during the cardiac cycle. However, for the case of circle of Willis, the arterial wall movement is very limited [55]. Hence, this assumption should not have much effect on the calculated flow field and the derived variables.

Regarding the oxygen transport, the following simplifications were made. Firstly, flux through the wall was assumed to be the same for the whole CoW—in reality, the flux differs, as it is highly dependent on the thickness of the arterial wall and its physical properties, which vary spatially [48]. Moreover, in all of the simulations, a wall-free model was assumed; therefore, no wall mass transfer resistance was present, and just the fluid mass transfer resistance was studied. Secondly, the calculation of Damköhler number was based on the properties from literature and not patient-specific ones. For exact calculations, it is necessary to gain this information from MRI together with the geometry and flow. Thirdly, we have been able to only validate the velocity field, not the oxygen distribution. To directly validate the presented results, the mass transfer of oxygen through the wall has to be measured by a non-intrusive technique. However, the resolution of state-of-the-art non-intrusive imaging techniques is very limiting, and currently, the changes in concentration of oxygen in the lumen and arterial wall are beyond their capabilities [56–59].

Finally, we have included only a single subject (healthy volunteer, 25 years old) in this study. To be able to draw statistically significant conclusions, a larger number of subjects should be assessed, including healthy volunteers and patients with different types of diseases. Nevertheless, the goal of our study was to present a framework for identification of hypoxia using subject-specific simulations.

## 5. Summary and conclusion

In the present study, we addressed the application of the combined MRI/CFD approach to provide detailed insights into the blood flow and corresponding transport of oxygen in the subject-specific brain vascular system. We considered two models of oxygen mass transfer: passive transfer, where the oxygen was treated simply as a dissolved chemical species in plasma, and active transfer, in which haemoglobin-bound oxygen was included in the model.

In the investigation, a subject-specific geometry of a part of the brain vascular system (circle of Willis) is analysed by a combined MRI/CFD approach. The flow conditions were specified to mimic the MRI measurements closely at the characteristic inlets and outlets. Detailed comparison of the blood flow field revealed an overall good agreement between 4D-flow MRI and CFD results. On the other hand, the comparison between CFD and MRI was less satisfactory in predicting the local distributions of the wall shear stress. The primary reason for this discrepancy was in the low spatial resolution of MRI in the proximity of the arterial wall. Finally, after getting the blood velocity fields, transport of oxygen was activated, and local concentration of the oxygen was calculated along the arterial walls. We showed that significant differences between the passive and active models of oxygen transport were obtained, not only in the magnitude of the non-dimensional arterial wall mass transfer obtained but also in their local distributions. This also confirmed the importance of using a more advanced model of oxygen transport in the subject-specific geometries of the brain vascular system. Finally, by comparing the local non-dimensional oxygen mass transfer at the arterial wall (Sherwood number) and corresponding non-dimensional rate of oxygen consumption (Damköhler number), we were able to identify the potential hypoxic regions within the cerebral vasculature. The Damköhler number was estimated to be in a range Da ⊂ 〈9; 57〉 for the case studied. Due to geometrical changes in the circle of Willis, several locations with the Sherwood number lower than the Damköhler number mentioned were found. This suggests the fluid phase limits oxygen transport to the wall. This information can be useful for follow-up patient studies dealing with early diagnosis or progression of dementia.

## Data Availability

The CFD results are available from the Dryad Digital Repository: https://doi.org/10.5061/dryad.4qrfj6qcx [60]. The data are provided in electronic supplementary material [61].
